# Radiological Differences in COVID-19 Related Lung Manifestations Between Smokers and Non-smokers: A Single-Center Retrospective Study in Jordan

**DOI:** 10.7759/cureus.38437

**Published:** 2023-05-02

**Authors:** Reem Hasweh, Ghaidaa S Khlaifat, Belal N Obeidat, Antoun A Khabaz, Mohammad B Ghanayem, Luna F Al-Zioud, Farah J Al-Dabbas, Samer A Al_Shbailat

**Affiliations:** 1 Department of Radiology, Al-Balqa Applied University, Al-Salt, JOR

**Keywords:** thoracic radiology, ground-glass opacity, smoking and covid-19, computed tomography (ct ), covid-19

## Abstract

Introduction

Despite the fact that smoking has been identified as a risk factor for respiratory diseases and lung infections, the relationship between smoking and coronavirus severity remains ambiguous. It is believed that smoking is a risk factor for pulmonary infections. However, the effect of smoking on COVID-19 patients is still controversial.

Objective

The aim of the study was to identify and analyze the distinct radiological features in COVID-19 patients with different smoking statuses. Additionally, the study sought to examine the association between smoking and the severity of pulmonary changes.

Methods

A retrospective cohort study of 111 patients who were referred to Al-Salt/Hussein Hospital, Al-Salt, Jordan, from January to June 2021, with a confirmed COVID-19 diagnosis and smoking status recorded. Patients' demographics, medical history, age, gender, comorbidity, and length of hospitalization were obtained from their medical records.

Results

Study groups were similar in median age, prevalence of chosen chronic diseases, and median length of hospital stay. Based on the median scores of the radiological findings in each lung lobe, no statistically significant differences were found between the scores and smoking status (p-values of >0.05; Mann-Whitney test).

Conclusion

Smoking is an independent risk factor for the severity of COVID-19. Smoking has no noticeable impact on interstitial manifestation in COVID-19 patients.

## Introduction

The highly contagious Coronavirus disease of 2019 (COVID-19) is a viral respiratory disease caused by the SARS-CoV-2 virus. It has evolved into a global pandemic affecting millions of people worldwide. The virus primarily affects the respiratory system, causing lung damage and other complications. Many studies have been trying to understand the risk factors that may make some individuals more vulnerable to severe COVID-19 symptoms than others [1]. Smoking has been identified as one such risk factor, and recent studies have shown that smokers are at a higher risk of developing severe COVID-19 symptoms than non-smokers [2,3].

Smoking is also a well-known risk factor for many respiratory infections and lung diseases, including chronic obstructive pulmonary disease (COPD) and lung cancer [4,5]. In addition to that, smoking can cause structural changes in the lungs, such as emphysema, which can lead to reduced lung function and increased susceptibility to respiratory infection [6]. Recent studies have shown that smokers are more likely to require hospitalization, mechanical ventilation, and are at a higher risk of dying from COVID-19 [7]. The mechanisms by which smoking increases the severity of COVID-19 are not fully understood.

As the pandemic continues to ravage communities, researchers and medical professionals have been working to understand the virus better and develop effective treatment methods. One area of interest is the radiological lung manifestations associated with COVID-19. Radiological imaging techniques, such as computed tomography (CT) scans, have been instrumental in identifying and monitoring the evolution of COVID-19-related lung damage [2]. Radiological differences between smokers and non-smokers with COVID-19-related lung damage have been the subject of recent research [8,9]. This is because smoking can cause a loss of lung elastic recoil pressure, leading to reduced lung function and increased amenability to respiratory infections, including COVID-19 [10].

A “case-control study” by Xie et al. conducted in China showed that patients with a history of smoking are more likely to have severe and widespread lung damage on radiological imaging than non-smokers [9]. This includes the presence of ground-glass opacities (GGOs), consolidations, and air bronchograms on chest CT scans, which are indicative of COVID-19-related lung damage. Therefore, extra support should be given to these patients [11].

On the other hand, a “case-control study” by Nikpouraghdam et al. aimed to investigate the differences in clinical, radiological, and laboratory findings between Iranian individuals who smoke and those who do not smoke with COVID-19 found that smokers with COVID-19 experienced significantly more chest pain and weakness and had lower white blood cell and neutrophil counts in their blood samples compared to non-smokers. However, there was no difference between the two groups in terms of lymphocyte count or CT scan findings. Additionally, the study observed a slightly lower mortality rate among smokers compared to non-smokers. Overall, the study sheds light on the impact of smoking on clinical outcomes in COVID-19 patients, but further research is needed to fully understand the relationship between smoking and COVID-19 [8].

By analyzing the radiological differences in lung manifestations between smokers and non-smokers, researchers hope to gain a better understanding of how smoking affects COVID-19 outcomes and develop targeted treatment approaches.

## Materials and methods

Study settings

We retrospectively conducted a search of the medical records of laboratory-confirmed COVID-19 patients (with a focus on their smoking status) at the Hussein/Al-Salt Hospital, Salt, Jordan, from January 24 to June 24, 2021.

The faculty of medicine's scientific research committee and the Institutional Review Board at Al-Balqa Applied University granted ethical approval, which waived the requirement for patients' informed consent.

Population

Information about patient demographics and medical history, such as age, gender, any pre-existing health conditions, length of hospitalization, and clinical presentation such as chest pain and shortness of breath was obtained from their medical records. Patients who were actively smoking or stopped after contracting COVID-19 were categorized as current smokers. To qualify as a current smoker, “the individual must have smoked at least 100 cigarettes in their lifetime and currently smoke cigarettes” (CDC, 2017) [12]. Another group was established, comprised of non-smoking COVID-19 patients who were admitted during the same timeframe. Non-smokers were defined as “patients who had either never smoked or had smoked fewer than 100 cigarettes in their lifetime” (CDC, 2017) [12]. 

Two methods were used to confirm the diagnosis of COVID-19: (1) isolation of COVID-19 and (2) positive results using real-time reverse-transcription polymerase chain reaction (RT-PCR) on nasopharyngeal swabs. Tracheal aspirates and oropharyngeal swabs were not used to confirm the diagnosis of COVID-19 at our institution.

The inclusion criteria set for the smoking group were: (1) patients who have a current smoking status or have quit smoking after being infected, (2) patients who have undergone CT scans, and (3) individuals whose ages range from 20 to 75 years. Patients who did not meet the following criteria were excluded, including (1) patients with abnormal radiological findings due to other pathological conditions such as lung cancer, pre-existing interstitial lung disease, or COPD, (2) COVID-19-positive patients with normal chest CT, or (3) cases with poor image quality. Finally, 45 patients with a current smoking history and 66 non-smoker patients were enrolled.

Imaging analysis

One radiologist (with a decade of expertise), who was blinded to both clinical data and smoking history, reviewed CT images. All images were observed on lung settings (width, 1500 HU; level, −700 HU). For each patient, a single CT scan which was performed on the first day of admission was analyzed after the diagnosis of COVID-19. The CT scan was evaluated according to a severity score which was proposed by Pan et al. [13] to define smoking-induced interstitial lung changes. Peripheral, bilateral, and basal predominant ground-glass opacities (GGOs) with or without consolidation were the characteristic chest CT findings in COVID-19 pneumonia. 

CT severity score was proposed by Pan et al. [13] which evaluates the lungs based on the involvement of each lobe. “Each of the five lung lobes was visually scored on a scale of 0 to 5 as follows: 0, no involvement; 1, < 5% involvement; 2, 5-25% involvement; 3, 26-50% involvement; 4, 51-75% involvement; and 5, > 75% involvement. The resulting global CT score was the sum of each lobar score from 0 to 25.”

Statistical analysis

GraphPad Prism software version 9.5.1, 2023 was used to perform statistical analyses. Counts, percentages, medians, and ranges were used to illustrate the results of descriptive analyses. Normality and lognormality tests were run, and the data of the radiological scores were found to be not normally distributed (Kolmogorov-Smirnov test, p-values were <0.05), and, accordingly, nonparametric tests were used. The Mann-Whitney test and Fisher’s exact test were used to investigate the presence of a relationship between variables. A p-value of <0.05 was regarded as statistically significant.

## Results

A total of 111 patients have been included in our study, of which 53.15% were females and 46.85% were males; 40.54% of the patients were smokers (Table 1). 

**Table 1:** Smoking status and participants’ radiological score in each pulmonary lobe, and the overall scores Hospital stay is displayed in the format: median (standard deviation). Scores are displayed in the format: median (range). Gender is displayed in the format: count (percentage of column total). *Fisher’s exact test. ** Mann-Whitney test.

	Non-smokers	Smokers	Overall	P-value
N (%)	66 (59.46%)	45 (40.54%)	111	
Gender				<0.0001^*^
Females	46 (69.70%)	13 (28.89%)	59 (53.15%)	
Males	20 (30.30%)	32 (71.11%)	52 (46.85%)	
Age				0.17702
Median	53	52	53	
Range	22-72	22-74	22-74	
Standard deviation	10.70	12.86	11.70	
Hypertension	17 (25.76%)	12 (26.67%)	29 (26.13%)	1.0000^*^
Diabetes	13 (19.7%)	12 (26.67%)	25 (22.52%)	0.4884^*^
Chronic kidney disease	1 (1.15%)	3 (26.67%)	4 (3.60%)	0.3935^*^
Hospital stay	6 (9.10)	6 (5.56)	6 (7.83)	0.9681^**^
Median scores (range)				
Score for the right upper lobe	2 (0 to 4)	2 (0 to 5)	2 (0 to 5)	0.6357^**^
Score for the right middle lobe	1 (0 to 4)	2 (0 to 5)	1 (0 to 5)	0.2656^**^
Score for the right lower lobe	2 (0 to 5)	2 (0 to 5)	2 (0 to 5)	0.6256^**^
Score for the left upper lobe	2 (0 to 4)	2 (0 to 5)	2 (0 to 5)	0.1893^**^
Score for the left lower lobe	2 (0 to 5)	2 (0 to 4)	2 (0 to 5)	0.2834^**^
Overall score	8 (0 to 22)	9 (0 to 24)	9 (0 to 24)	0.3608^**^

The individuals participating in the study were categorized into two groups according to their smoking status: smokers and non-smokers (Table 1). The difference in gender between the two groups was statistically significant (p-value of <0.0001; Fisher’s exact test); most non-smokers were females (69.70%), and most smokers were males (71.11%).

Study groups were similar in median age and in the prevalence of chosen chronic diseases, including chronic kidney disease, hypertension, and diabetes. Smokers and non-smokers also stayed at the hospital for the same median day.

The median scores based on the radiological findings in each lung lobe are illustrated in Table 1, along with the minimum and maximum values of each score. No statistically significant differences were found between the scores and smoking status (p-values of >0.05; Mann-Whitney test).

## Discussion

We analyzed and evaluated the radiological features of 111 individuals who had been diagnosed with COVID-19 with different smoking statuses. The typical CT findings of COVID-19 in both study groups (smokers and non-smokers) demonstrated GGO or mixed GGO and consolidation. Lesions were predominantly peripheral, and most COVID‐19 patients showed bilateral abnormalities. These findings were well-established by previous studies [14,15] and were consistent with ours.

Our study demonstrated that there is no significant association between smoking and severe interstitial manifestations on CT images. Our findings aligned with two systematic reviews that indicated smoking had no significant impact on the outcomes of COVID-19 patients. This can be attributed to the complex interaction between Coronavirus and smoking. Although smoking may enhance the expression of angiotensin-converting enzyme 2 receptors, expose patients to harmful chemicals, and increase their risk of contracting numerous diseases, its nicotine content may inhibit inflammatory reactions, thus counteracting the harmful effects of smoking for COVID-19 patients [16,17].

Contrary to that, a study of 86 patients in Hunan, China, conducted by Xie et al. found that patients with a history of smoking exhibited more severe interstitial features and that these patients may have less easily differentiated margins and more residual lesions on follow-up CT scans. In the same study, however, it was shown that non-smokers demonstrated larger lung involvement on chest CT images, but with well-defined margins of ground-glass opacities (GGOs), indicating that the lesions were continuously being resorbed, as was shown in follow-up CT scans [9].

On the same side, another study established by the Turkish Ministry of Health, which retrospectively evaluated the thorax CT scan for 121 patients by two radiologists according to the involvement of opacities in each region of the lung which were divided into 20 regions (10 regions in each lung), found that the CT scans of both active and former smokers showed higher severity scores than the non-smokers [18].

Additionally, a retrospective study conducted by Piasecki et al. in Wisconsin, United States of America, reported findings that support the protective effect of smoking in COVID-19 patients and revealed favorable potential effects for nicotine replacement therapy (NRT) for current smoker COVID-19 patients where the mortality was less than current smokers without NRT prescriptions [19]. The same study also showed that the vaccination efficacy was influenced by the smoking status, where the mortality rate and ICU admissions were lower in smokers than non-smokers in correlation with the vaccination status [19]. These results would support the protective idea of smoking in COVID-19 patients.

To the best of our knowledge, this article is the first to evaluate chest CT features in the different smoking statuses of COVID-19 patients in the Arab region. Our conclusions are convincing, and the analysis is rigorous. This study also has limitations. First, because our study is retrospective, it depends on the review of charts that were not originally designed to collect data for research purposes. Also, some information is bound to be missing. Second, the small sample size may have affected the strength of this study. Specifically, the smoking group consisted of a relatively small population due to the hospital's limited number of reported cases. Third, the study was single-centered, which made our conclusion less representative. Finally, among the patients included in our study, most of the current smokers were males. This is explained by the gender disparity in smoking rates in Jordan, with males being more likely to smoke than females. One possible explanation for this phenomenon is the social stigma that surrounds female smoking [20].

## Conclusions

Contrary to popular assumptions that smoking causes more severe disease in COVID-19 patients, our findings indicated that there were no significant differences in the clinical presentation of COVID-19 patients between smokers and non-smokers. However, due to the above limitations, studies with larger sample sizes and more rigorous designs should be performed.
